# Functional traits, the phylogeny of function, and ecosystem service vulnerability

**DOI:** 10.1002/ece3.601

**Published:** 2013-07-30

**Authors:** Sandra Díaz, Andy Purvis, Johannes H C Cornelissen, Georgina M Mace, Michael J Donoghue, Robert M Ewers, Pedro Jordano, William D Pearse

**Affiliations:** 1 Instituto Multidisciplinario de Biología Vegetal (CONICET-UNC) and FCEFyN, Universidad Nacional de CórdobaArgentina; 2 Department of Life Sciences, Imperial College LondonSilwood Park, SL5 7PY, United Kingdom; 3 Systems Ecology, Department of Ecological Science, Faculty of Earth and Life Sciences, VU UniversityAmsterdam, The Netherlands; 4 Department of Genetics, Evolution and Environment, University College LondonGower Street, London, WC1E 6BT, United Kingdom; 5 Department of Ecology and Evolutionary Biology, Yale UniversityNew Haven, Connecticut; 6 Integrative Ecology Group, Estación Biológica Doñana, CSICSevilla, Spain

**Keywords:** Biodiversity loss, ecosystem vulnerability, functional traits, phylogenetic conservatism, specific effect function, specific response function

## Abstract

People depend on benefits provided by ecological systems. Understanding how these ecosystem services – and the ecosystem properties underpinning them – respond to drivers of change is therefore an urgent priority. We address this challenge through developing a novel risk-assessment framework that integrates ecological and evolutionary perspectives on functional traits to determine species’ effects on ecosystems and their tolerance of environmental changes. We define Specific Effect Function (SEF) as the per-gram or per capita capacity of a species to affect an ecosystem property, and Specific Response Function (SRF) as the ability of a species to maintain or enhance its population as the environment changes. Our risk assessment is based on the idea that the security of ecosystem services depends on how effects (SEFs) and tolerances (SRFs) of organisms – which both depend on combinations of functional traits – correlate across species and how they are arranged on the species’ phylogeny. Four extreme situations are theoretically possible, from minimum concern when SEF and SRF are neither correlated nor show a phylogenetic signal, to maximum concern when they are negatively correlated (i.e., the most important species are the least tolerant) and phylogenetically patterned (lacking independent backup). We illustrate the assessment with five case studies, involving both plant and animal examples. However, the extent to which the frequency of the four plausible outcomes, or their intermediates, apply more widely in real-world ecological systems is an open question that needs empirical evidence, and suggests a research agenda at the interface of evolutionary biology and ecosystem ecology.

## Introduction

Human well-being largely depends on benefits derived from ecological systems known as ecosystem services. However, anthropogenic drivers of change are having widespread effects on ecosystems, potentially compromising their ability to continue to provide these benefits (Millennium Ecosystem Assessment 2005, Cardinale et al. 2012). Understanding risks to ecosystem services presents a formidable intellectual challenge to ecologists, conservation scientists, and evolutionary biologists. Here, we integrate recent ecological research on functional traits of organisms with phylogenetic comparative approaches and show how the two approaches together provide new ways to refine assessments of the vulnerability of ecosystem properties and services in the face of environmental change.

From the perspective of functional ecology, functional traits (Box 1) are known to underpin both species’ contributions to ecosystem properties and services and their tolerance to environmental stressors and disturbances (Suding et al. 2008). Crucially, however, species’ contributions and vulnerabilities will often depend not on individual functional traits but on *combinations* of traits. In parallel, a phylogenetic perspective has been widely used in functional trait studies at levels ranging from individuals to communities (Webb et al. 2002; Cavender-Bares et al. 2009, 2012; Pausas and Verdu 2010; Pillar and Duarte 2010; Srivastava et al. 2012). The potential usefulness of a phylogenetic perspective for studying ecosystem properties has also been recognized for some time (Edwards et al. 2007; Cavender-Bares et al. 2009; Matthews et al. 2011; Gravel et al. 2012), but work to date has focused mostly on individual traits, and connections between phylogenies and ecosystem services have only recently been proposed (Faith et al. 2010; Srivastava et al. 2012). Here, we introduce evolutionary concepts to the risk assessment of ecosystem properties by applying phylogenetic tools to integrative measures of species’ effects on ecosystem processes and their responses to environmental drivers.

**Box 1. Key definitions in the functional trait approach**

**Functional traits** are morphological, biochemical, physiological, structural, phenological, or behavioral characteristics that are expressed in phenotypes of individual organisms and are considered relevant to the response of such organisms to the environment and/or their effects on ecosystem properties (Violle et al. 2007). This crucial position of functional traits at the crossroads between responses to the environment and ecosystem properties explains the increasing attention given to them by both evolutionary biologists and functional ecologists. This duality is reflected in the literature by distinguishing between effect and response traits (Díaz and Cabido 2001; Lavorel and Garnier 2002; Naeem and Wright 2003; Suding et al. 2008).

**Effect traits** of a species underlie its impacts on ecosystem properties and the services or disservices that human societies derive from them (Aerts and Chapin 2000; Grime 2001; Lavorel and Garnier 2002), whether or not such traits represent an adaptive advantage to the individual itself. Examples of effect traits include water retention capacity in bryophytes (regulating ecosystem hydrology), leaf nitrogen content in vascular plants (accelerating nutrient cycling rate), and burrowing behavior (altering soil structure) or gut digestive features (influencing nutrient turnover) in animals (more examples, with references, are given in Table S1A).

**Response traits** influence the abilities of species to colonize or thrive in a habitat and to persist in the face of environmental changes. Plant examples include seed size (related to recruitment capacity under different disturbance regimes), bark thickness (conferring fire tolerance), and leaf size (leading to different heat balances). Animal examples are bill shape and size (allowing the capture of food items of different kind and size) in birds, desiccation tolerance in soil arthropods, and tongue length (giving access to nectar contained in flowers of different size and shape) in pollinators (see Table S1B for more examples and references). The same trait may in some cases act as both response and effect traits (Suding et al. 2008): for instance, leaf nitrogen content in plants and body size in animals both underlie multiple responses to the environment and effects on ecosystem properties (Table S1A, B).

Traits can be the joint expression of underlying biophysical and biochemical properties and processes of an organism; whether a trait is a combination of such properties or itself one of such properties is a question of judgment and objective of the study. For example, leaf toughness can be seen as a trait that depends on anatomical characteristics such as venation architecture and density and chemical characteristics such as lignin concentration, or lignin concentration itself can be considered a trait. In this article, we take a broad view of traits without specification of whether or not they can be deconstructed into simpler characteristics. The relevance of functional traits in species’ response to the environment or species’ effect on ecosystems is usually established empirically by observation or manipulation of the ecosystem under study or by extrapolation from other studies. Examples of characteristics or suites of properties commonly regarded as effect or response traits can be found in Table S1.

### Organismal traits, ecosystem function, and vulnerability to environmental change

The erosion of the world's biota by environmental change carries a dual risk. One risk is that the continuing delivery of ecosystem services may be compromised as the ecosystem functions and processes regulated by biotic communities are altered (Cardinale et al. 2006; Worm et al. 2006; Estes et al. 2011). The other is the risk of local or even global loss of species whose tolerance is surpassed (Butchart et al. 2010; Secretariat of the Convention on Biological Diversity 2010; Larigauderie et al. 2012), and the associated reduction in evolutionary capital — the ability of evolutionary processes to deliver future benefits to society (Faith et al. 2010). These risks emphasize different aspects of living organisms: the continuity of species’ contribution to ecosystem properties and services, and their capacity to survive and thrive in the face of changing selective pressures. Interactions between these two aspects lead to the identification of two concepts central to our framework for predicting vulnerability of ecosystem properties and benefits to environmental change: Specific Effect Functions and Specific Response Functions. Here, we use the word “function” in its original sense, that is, to “execute or perform”. “Specific function” refers to the “execution” of an effect of a species on a given ecosystem property and also to its “performance” in the face of given environmental drivers. The terms Specific *Effect* and *Response* Functions are used to distinguish these two aspects, in keeping with the already widespread concepts of functional effect traits and functional response traits (Lavorel and Garnier 2002; Naeem and Wright 2003; Suding et al. 2008).

**Figure 1:**
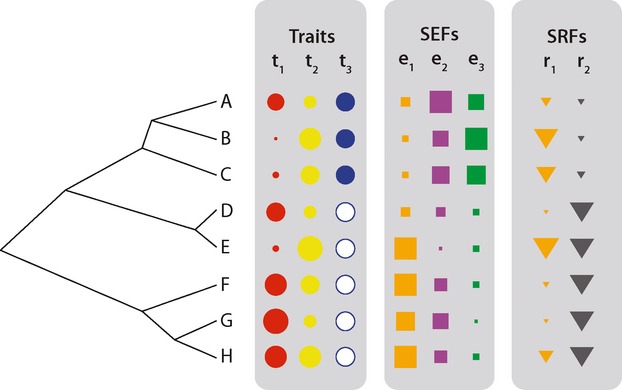
Phylogeny, traits, and effect and response functions. A–H are species related as shown by the phylogenetic tree on the left. In the first rectangle to the left, traits t_1_ and t_2_ are continuous variables (e.g., specific leaf area; sizes of symbols indicate species’ values of these traits); whereas t_3_ can take either of two states (e.g., does or does not fix nitrogen). In the second rectangle, Specific Effect Functions (SEFs, e_1_ to e_3_) are functional effects of species on the ecosystem; examples include decomposability, long-distance dispersal of seeds, and production of bushmeat. Each SEF's color indicates which traits determine its value (e.g., the orange e1 is the product of t_1_ (red) and t_2_ (yellow)). Because SEFs are not the same as traits, they can show different phylogenetic patterns: e_1_, for example, shows a much stronger phylogenetic signal than either of the traits that influence it. In the third rectangle, Specific Response Functions (SRFs, r_1_ and r_2_) indicate each species’ tolerance of different drivers of change (e.g., habitat fragmentation). The traits determining SRFs and SEFs could be the same (e.g., r_1_ and e_1_) or different (e.g., r_2_ and e_1_). A strongly patterned SRF - like r_2_ - means that the driver could cause the loss of whole clades from the ecosystem; if an SEF also shows strong signal, this loss of clades may reduce the range of SEF values (e.g., e_1_ and e_3_) and lead to the loss of all large-effect species (e.g., e_3_).

A *Specific Effect Function (SEF)* is the per-unit capacity of a species to influence an ecosystem property or service. The relevant units will depend on the organism and the ecosystem property or service in question: for example, number of seeds dispersed per individual in birds, volume of water transpired per unit leaf area per unit time in trees, and amount of a heavy metal absorbed per unit length of hyphae in mycorrhizas. SEF is equivalent to *E* in Suding et al.'s (2008) response–effect model for plants, on which our framework builds. In other words, an SEF is the difference made to a particular process at the ecosystem level by a standard “amount” of a species. An SEF depends on one or more functional traits of the species; such traits are termed functional effect traits (Box 2, Fig. 1). An example of an SEF is the capacity of some rodents and ungulates to redistribute nutrients over the landscape and thereby affect resource availability for other organisms; this SEF – which could be expressed as, for example, amount of nitrogen and phosphorus redistributed per individual vole or rhinoceros per month – is underpinned by a set of effect traits including body size, mobility-related characteristics such as limb anatomy, and behavioral traits such as defecation patterns, territorial displays, or solitary versus colonial habit. Although the traits that underlie an SEF are often linked to survival and reproduction and therefore under natural selection, the SEF itself does not need to be. Rather, SEFs are often a side effect of selection on traits that affect fitness. In the example above, the nutrient redistribution capacity (the SEF) may not itself directly link to rodent or ungulate fitness, although most or all the underlying traits presumably do. The same effect trait will often influence more than one SEF; for example, leaf nitrogen content influences plant relative growth rate, palatability to herbivores and litter decomposability (Aerts and Chapin 2000) (see Table S1A for further examples of SEFs and the effect traits that underpin them, with references). Note that although we mostly deal with ecosystem *services*, that is, species effects on ecosystem properties that are *beneficial* to people, the concept of SEFs can be equally applied to ecosystem *disservices*, that is, ecosystem properties that are detrimental.

A *Specific Response Function (SRF)* is the ability of a species to maintain or enhance its quantity in response to a specified change in the abiotic or biotic environment, or to invade this environment afresh. SRF is measured as the proportional change in the biomass of the species – for which changes in ground cover or population size will often be good proxies – per unit of change in an environmental driver. SRF is strongly rooted in the concept of *Rj* set out by Suding et al. (2008), but goes further by explicitly distinguishing between species’ responsiveness (SRF) and species’ realized response to environmental drivers, the latter depending both on SRF and the specific environmental context. SRF is also analogous to, but broader than, the concept of elasticity or proportional sensitivity sensu Caswell et al. (1984) and Caswell (2000); for instance, SRFs can be applied readily to multispecies assemblages in ecosystems. An SRF typically depends on some combination of underlying *functional response traits* (Box 1, Fig. 1). Typical SRFs are the tolerances of species to different abiotic stresses (e.g., frost, drought, pollution, low nutrient availability), biotic pressures (e.g., competitors, pathogens, herbivores, predators, parasites), or particular disturbance regimes (e.g., fire); high values of an SRF indicate that the species is able to tolerate the regime well, whereas low values indicate that it is susceptible. Examples of SRFs illustrate the concept: drought tolerance in vascular plants (SRF) may depend on several response traits including xylem vessel diameter, leaf phenology, chemical and physiological traits, root depth, and water storage adaptations; the capacity of angiosperms to persist in the face of repeated aboveground disturbance in mesic and cold climates (SRF) depends on the capacity of their seeds to get buried quickly and form a persistent soil bank, which in turn is well correlated with the response traits seed size and shape; and the ability of vertebrate species to withstand harvesting by humans (SRF) depends on traits such as elusiveness, poor palatability, or rapid reproductive rates. See Table S1B for references and more examples. Comparative data on realized responses may be our most direct information about species’ SRF values, but can only be safely compared at face value if all species have experienced the same intensity of drivers. Otherwise, responses must be modeled as a function of driver intensities, with the residuals reflecting SRF values.

Importantly, there is no reason to expect one-to-one mapping between SEFs and effect trait values, or between SRFs and response trait values. This is because a given SEF may be achievable through many different combinations of effect traits and their values; the same is true of SRFs and response trait values (Fig. 1; see Wainwright 2007 for a detailed exposition in the context of morphometric traits). For instance, leaf litter decomposability and palatability to herbivores are SEFs that depend on combinations of leaf toughness and concentrations of nutrients, lignin, polyphenols, specific toxins, and leaf cuticle properties. Physically tough leaves (e.g., in Proteaceae) and juicy, nutritious leaves with powerful toxins (e.g., tropane alkaloids in Solanaceae) will both be unpalatable to a broad range of herbivores, affecting the ecosystem's carrying capacity for animals. Likewise, the capacity to persist in the face of drought and fire is an SRF that can be produced using different strategies: some plants have leaves that are structurally or physiologically resistant to drought; others instead have dormant buds that resprout after the leaves have been lost, using resources stored in deep belowground organs; while a third strategy is to regenerate from seed. Populations with a low SRF under environmental change will experience directional selection for higher SRF values, which could arise through multiple combinations of changes in functional trait values.

The same traits may underpin both SRFs and SEFs (Lavorel and Garnier 2002; Suding et al. 2008; Lavorel and Grigulis 2012; Luck et al. 2012); also compare traits listed in sections A and B of Table S1). For instance, a large body size of birds and mammals influences both their capacity to disperse seeds and to be good providers of meat protein (SEFs), but will be correlated with their susceptibility to decline in the face of hunting (SRF) (see case study below). Similarly, a large body size of litter-eating invertebrates such as woodlice relates both to how efficiently they fragment litter, thereby accelerating its decomposition (SEF), and to their desiccation tolerance (SRF). Furthermore, what constitutes a relevant SEF is context dependent. For example, the capacity of predators to catch particular prey types is an SEF, if the interest is in maintaining primary productivity in the presence of herbivores, but the same capacity can be seen as an SRF if the focus is on protecting an endangered predator in the face of decreasing prey numbers.

The extent to which a species contributes to ecosystem properties and services at the scale of local plots or patches contained in a landscape will often (though not always) depend strongly on the species’ local abundance (see Box 4). However, the contribution can only be made if the species is present. In other words, focal community- and ecosystem-level properties (linked to SEFs) can be strongly limited by species availability in and recruitment from the species pool (Symstad and Tilman 2001; Naeem and Wright 2003; Zobel et al. 2006) – and these are directly governed by SRFs, rather than SEFs. Our focus in this article is on this broader scale: how environmental changes interact with species’ SRFs to shape the suite of species and thence the SEF values that persist and so *can* contribute to ecosystem properties and services.

In the next section we show that both the correlation of SEF with SRF across species and the extent to which each of them tends to be similar among closely related species are important in assessing the risk that environmental change will impact the relevant species pool and thus the potential to deliver ecosystem properties and benefits to people. We then explore – using case studies from different systems – how these functions, traits, and phylogenetic signals relate to each other and to the vulnerability of ecosystem properties and benefits. The relevant species pool is defined by the scale of the ecosystem property or service of interest. In this article we focus at the local (i.e., landscape-level) species pool because most of the ecosystem properties and benefits we discuss occur at the level of local plots or patches in a landscape mosaic. The relevant pool would be different, however, if the ecosystem property or benefit of interest operates at a broader scale (e.g., the heat exchange with the atmosphere that underpins regional weather patterns, or the provision of corridors needed for large mammal migration).

### Linking traits, functions, and phylogeny

Functional traits are aspects of organismal phenotypes and have evolved along the branches of phylogeny. Because evolutionarily closely related species tend to be ecologically similar and respond similarly to selection, functional trait values are likely to show phylogenetic signal – that is, they will tend to be more similar in closely related species than in distant relatives (Harvey and Pagel 1991; Freckleton et al. 2002; Ackerly 2009). Note that this argument does not mean that close relatives *have* to be similar – adaptive radiations show that change can be rapid when lineages adapt to new niches (Gavrilets and Losos 2009; Yoder et al. 2010) – just that they will *tend* to be similar. However, because SEFs and SRFs reflect *combinations of multiple functional traits*, they can display a very different tempo and mode of change from those seen in the traits themselves. In the simplest case, a function may be the same in all species within part of a clade (strong phylogenetic signal and a low inferred rate of change), whereas the underlying traits that confer that function might take different values in different species and show weak phylogenetic signal and apparently rapid change. For example, the use of birds as the predominant seed disperser might characterize an entire family of plants, despite multiple independent evolutionary shifts within that family in traits that make this mode of dispersal possible, such as the color of the fruit used to attract birds. Conversely, a function may show a much weaker phylogenetic signal and faster evolution than its constituent traits. As an illustration, seed dispersal by birds might have arisen independently in many different plant lineages (weak phylogenetic signal and a rapid overall rate of change) depending on the evolution of different underlying characters which, themselves, may evolve slowly and show strong phylogenetic signal. For example, all the species in one lineage might present their seeds in drupaceous fleshy fruits, whereas the species in another lineage might attract birds by having colorful seeds.

**Figure 2:**
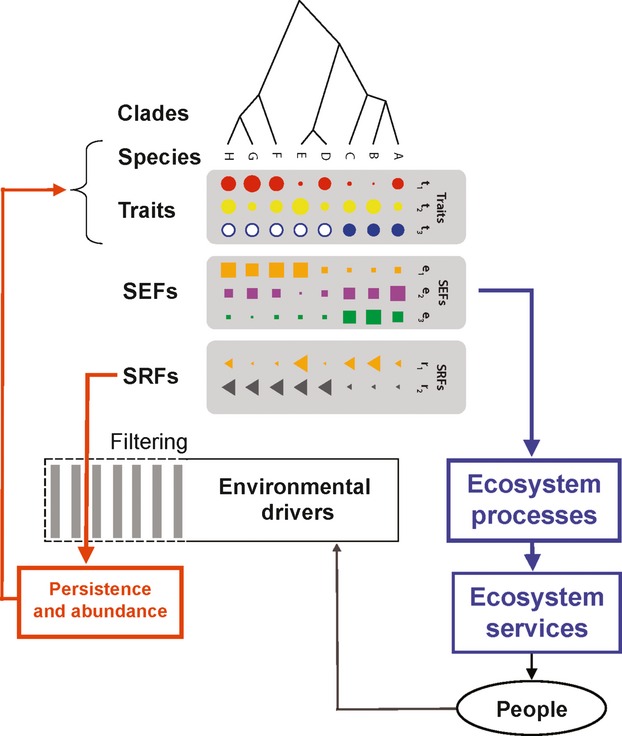
Functional trait evolution, ecosystem function, and environmental change drivers. Species or populations in a given landscape have certain functional trait values that determine their specific effect functions (SEFs) and their specific response functions (SRFs) to different environmental drivers (see Fig. 1 for explanation of symbols). Within a given environmental envelope, the SEFs determine the potential of the species pool to deliver functions. These, together with species’ local abundance, influence ecosystem properties, which are the basis of many ecosystem benefits or detriments perceived by people (blue pathway). SEFs can also influence ecosystem benefits directly. Ecosystem benefits contribute to human well-being, which, together with a complex array of socioeconomic aspects, influence environmental drivers of ecological change. These drivers (e.g., altered frequency of frost, drought, fire, or soil disturbance; higher nitrogen loading; or invasion by new predators, competitors or pathogens) exert a filtering process on the species pool according to the latter's SRFs, that is, they affect the landscape-scale persistence and the local abundance of species or populations that are susceptible or resistant to them (red pathway). Other links between external drivers and ecosystem properties (such as direct regulation of decomposition or evapotranspiration by temperature), or between drivers and society, are acknowledged but not displayed because they are not central to the main argument of this article.

Combining SRFs and SEFs integrates the evolution of functional traits, ecosystem properties and services, and their vulnerability to environmental change drivers into a single framework (Fig. 2). Whereas ecosystem properties and services are underpinned by the distribution of the SEFs, it is the SRFs that are the targets of selection and sorting by environmental drivers. The future potential species complement of any ecosystem is therefore directly related to the SRFs of the species within the source pool rather than their SEFs; the SEFs and the resulting (dis)continuity of ecosystem benefits to people are side effects of this process of environmental filtering and selection. Drivers of change will tend to eliminate those species in the species pool whose SRFs leave them susceptible, while promoting the invasion and expansion of species with high SRFs under the new environmental conditions. However, the properties of, and the services from, the resulting assemblage of species will depend not on the SRF but mostly on the SEF. These different causes and consequences of changes to SEFs and SRFs make patterns of association between them of particular importance for predicting the ecosystem-level consequences of change.

### Risk assessment for ecosystem properties and services

The correlation between SEFs and SRFs and their phylogenetic distributions will influence the potential of the ecosystem to deliver function under different environmental changes. Any ecosystem property or benefit is at risk from a driver of change if the species whose SEFs underpin the property have SRF values that make the species susceptible to that driver. We argue that a system is likely to be more vulnerable to disruption whenever an important range of SEF values is provided solely by members of a single clade (set of species that are each other's closest relatives) in the local species pool, rather than by members of multiple, independently evolved clades. This argument assumes that distantly related species providing a given range of SEF values are more likely than close relatives to differ in their values of SRFs and the traits that underpin them. Thus, even if one lineage conferring the function is lost, others may remain, which would safeguard the potential of the species pool to provide the function. If we knew the susceptibility of each species in the pool directly, we might not need the phylogenetic information; however, in the absence of such complete information, the knowledge that the functionality evolved separately in distantly related lineages can help us gauge the vulnerability of the system. The phylogeny also provides a basis for generalizing to unstudied or poorly known members of the relevant clades: if all the well-studied members of a clade are known to be susceptible, then unstudied members may be inferred to be similarly susceptible (see, e.g., Willis et al. 2008). In the absence of phylogenetic knowledge there would be little basis for such generalizations.

**Figure 3:**
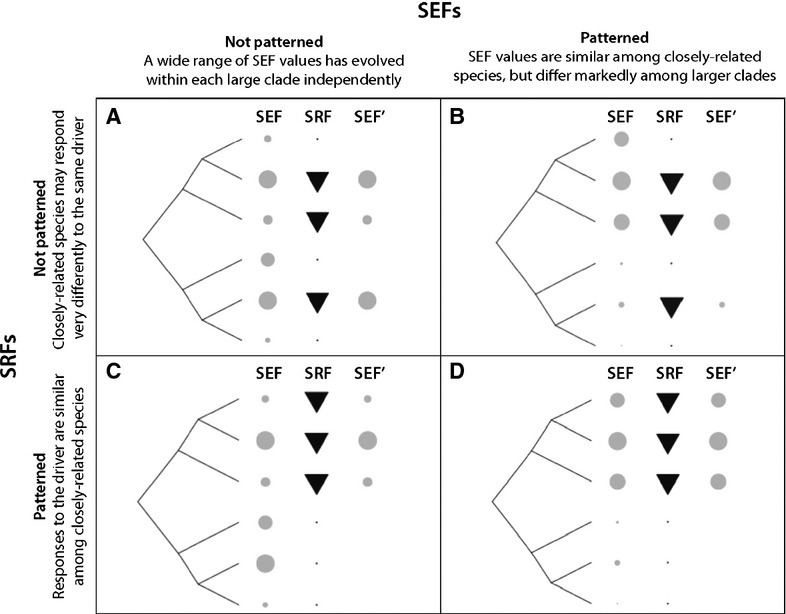
The ecosystem-level consequences of phylogenetic patterning of effect and response functions in the species pool. Values of species effect function (SEF) are indicated by the sizes of circles, and values of species response function (SRF) by the sizes of triangles; both of these are likely to be shaped by multiple underlying functional traits. Species with larger triangles are more tolerant of the driver of environmental change being considered. The driver removes less tolerant species or otherwise prevents them from expressing their effect. How well the resulting spectrum of effect functions (SEF’) in the remaining species reflects their original distribution (SEF) depends on the phylogenetic pattern of both SEF (columns in the Figure) and SRF (rows) and on the correlation between SEF and SRF. Each of the four cases, A–D, is discussed in the text.

The extent to which an SEF and SRF are correlated and show phylogenetic signal can help to provide a risk assessment for ecosystem-level functionality. Box 2 depicts four extreme situations, from minimum concern when SEF and SRF are neither correlated nor show a phylogenetic signal to maximum concern when they are negatively correlated (i.e., the most important species are the least tolerant) and phylogenetically patterned (lacking independent backup). The frequency of the four plausible outcomes in real-world ecological systems is an open question that needs empirical evidence. Many functional traits show some degree of phylogenetic signal (Freckleton et al. 2002; Ackerly 2009), which seems to suggest that Figure 3D could be more prevalent than Figure 3A. However, other traits show only weak phylogenetic signal (reviewed by Srivastava et al. 2012) and, as argued above, SEFs and SRFs may anyway show different phylogenetic patterns from their underlying traits, so the question remains open. Comparative analyses of how species respond to particular drivers show a range of outcomes. Global mammalian IUCN Red List data show strong phylogenetic signal for extinction risk from overexploitation (mediated by life-history traits that themselves show very strong phylogenetic pattern), but only moderate signal from habitat loss (Fritz and Purvis 2010). Sensitivity to disturbance repeatedly shows strong phylogenetic pattern in lake zooplankton communities (Helmus et al. 2010). However, Thuiller et al. (2011) report that projected responses to climate change in European mammals, birds, and plants show only weak phylogenetic signal. Furthermore, there are at least two reasons why SEFs and SRFs may not generally be as strongly patterned as individual functional traits. The first is that any ecosystem contains only a small, usually nonrandom, sample of the global set of species: functional trait values may be strongly patterned in the global phylogeny, but random or nearly so within the phylogeny of species at smaller spatial scales. Second, the mapping between functional trait and function need not be simple: as discussed previously, the phylogenetic signal of SEFs and SRFs can differ from that of the underpinning traits.

**Box 2. Phylogeny of Function and Vulnerability to Environmental Change Drivers**

When considering an ecosystem's potential to produce a given property or service when faced with a given driver of change, the outcome depends on the correlation and phylogenetic patterning of SEF and SRF, as shown in Figure 3. There are four extremes in terms of phylogenetic patterning, as listed below Figure 3:

- Neither SEF nor SRF shows phylogenetic signal. The environmental change driver will lead to the loss of a phylogenetically random set of species, in which case the overall amount of evolutionary history in the local species pool will largely be maintained under environmental change (Nee and May 1997). If the SEF and SRF are strongly negatively correlated, such that species contributing most to the process are also the most sensitive, then the function is at risk. If SEF and SRF are uncorrelated, the capacity to provide the function will be resistant, as the distribution of SEF values after the driver has operated (SEF′ in Fig. 3A) will be a random sample of the initial SEF distribution, with a similar mean and range.
- The SEF shows strong phylogenetic signal, but the SRF does not (Fig. 3B). In such cases, the capacity to provide the function is resistant. The fact that SEFs and SRFs differ greatly in their phylogenetic patterning precludes a strong correlation between them, so the distribution of SEF values remaining after the driver has operated will be a random sample of the initial spectrum, with a similar mean and range. Additionally, no major clades are removed by the driver, so the overall amount of evolutionary history will be largely maintained unless species loss is extremely high (Nee and May 1997).
- The SEF shows no phylogenetic signal, but the SRF shows strong signal (Fig. 3C). Again, the mismatch between phylogenetic signal strength means that the capacity to provide the function is retained. However, the signal in the SRF means that whole clades are likely to be lost with a corresponding reduction in overall evolutionary history in the local species pool, arguably conferring an increased vulnerability to other drivers of change.
- SEF and SRF both show strong phylogenetic signal (Fig. 3D). The overall evolutionary history in the local species pool will be strongly reduced, as in Figure 3C. The impact on the SEF distribution depends on the correlation between SEF and SRF across the species in the source pool from which the community is drawn. A strong correlation leads to a reduction in the range of SEF values and to a possible loss of function (depending on whether the part of the SEF range that is removed is important for function).

Correlations between SEF and SRF can be assessed either phylogenetically or nonphylogenetically. In terms of the outcome for a particular system or set of systems, the nonphylogenetic correlation is the more important: it does not matter whether the SEF and SRF have changed together through phylogeny, just whether species with high SEF values have SRF values that confer high susceptibility. However, studies aiming to make more general predictions will sometimes benefit from a phylogenetic analysis, particularly when focusing on evolutionary questions. We thus report both phylogenetic and nonphylogenetic correlations between SEFs and SRFs in our case studies.

### Applying the framework: case studies

**Figure 4:**
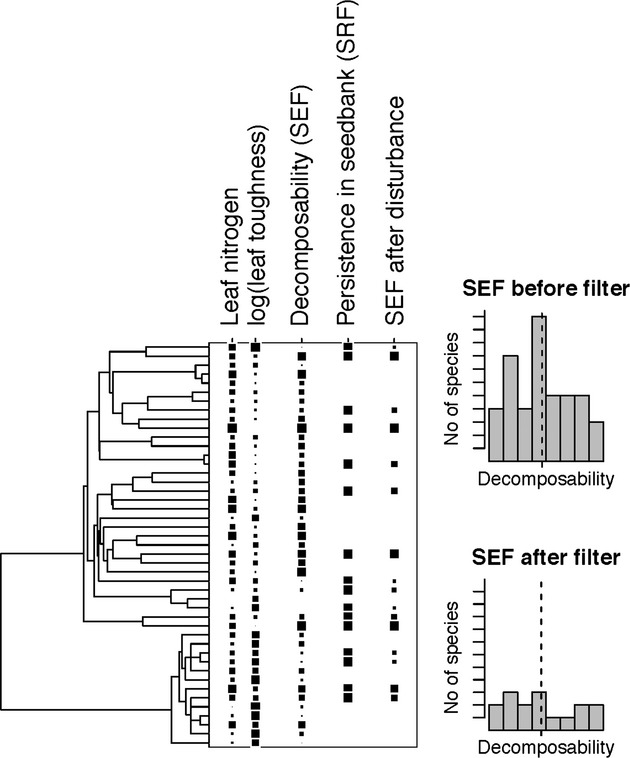
Decomposability and changing land use in Northern England. The contribution of highly decomposable litter is a key plant SEF for nutrient cycling and the ecosystem service soil fertility. Decomposability of litter, measured experimentally (Cornelissen 1996), shows moderate phylogenetic signal (λ = 0.49) in the angiosperm flora of Sheffield, United Kingdom. In a phylogenetic comparative analysis (using phylogenetic GLS; Freckleton et al. 2002), 52.0% of the variance in decomposability among species is explained by leaf nitrogen concentration and leaf toughness; neither leaf phosphorus concentration nor specific leaf area explained significant additional variance (both *P* > 0.05). The moderate phylogenetic signal of the SEF results from weak signal in one of the functional traits (leaf nitrogen concentration, λ = 0.16) and strong signal in the other (leaf toughness, λ = 0.71). We consider how clearance of the vegetation and disturbance of the soil might be expected to impact on decomposability. Species that persist in the soil seed bank can tolerate such major disturbance. We used tolerance to disturbance as an SRF, using the product of log (seed mass) and a seed shape measure as a proxy for species’ ability to persist in the seed bank (Thompson et al. 1993). This SRF shows only weak phylogenetic signal (1−D = 0.13) among these species, in contrast to the SEF. The difference in phylogenetic pattern makes strong correlation between SEF and SRF unlikely, and indeed the correlation is negligible (both phylogenetic and nonphylogenetic *r*^2^ << 1%; quadratic regressions are also nonsignificant). Consequently, even though only 16 of the 45 species can tolerate disturbance, the distribution of the SEF is almost unaffected – the full range of fast, medium, and low decomposabilities in the species pool is maintained (compare upper and lower histograms), and the mean and standard deviation (not shown) hardly change. The ecosystem function and societal benefit, nutrient cycling, and soil fertility are thus predicted to remain largely unchanged. This example corresponds most closely to the upper right corner of Figure 3.

**Figure 5:**
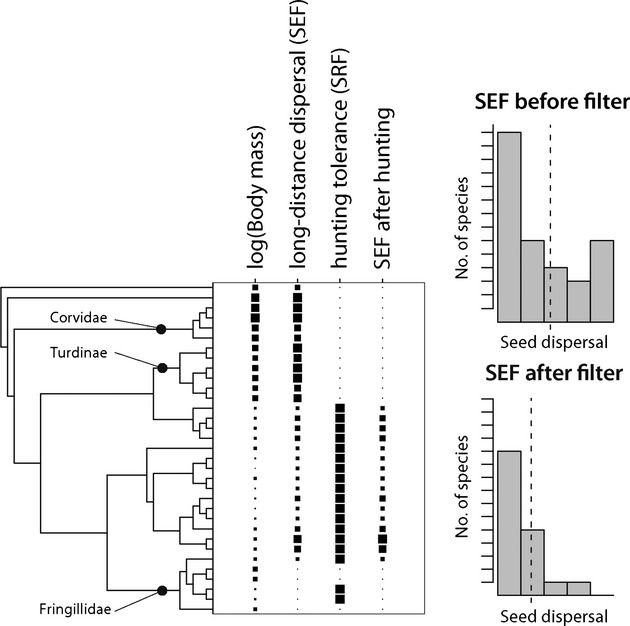
Long-distance seed dispersal and susceptibility to population decline due to hunting in the European Mediterranean area. Long-distance dispersal is a key bird SEF that contributes to the maintenance of plant metapopulations and colonization of new patches during range expansion. Woody fleshy-fruited plants in a Mediterranean ecosystem rely on birds for dispersal over long distances, and bird species differ markedly in how much long-distance dispersal they are observed to provide (data from Jordano et al. 2007 and references therein). These plants benefit humans by supporting seminatural agroecosystems with a key role in local socioeconomies (Zamora et al. 2010). This SEF shows strong phylogenetic signal (λ = 1; test vs. zero, *P* << 0.001). Larger species such as corvids (Corvidae) and thrushes (Turdinae) provide much of the long-distance dispersal, whereas smaller species like finches (Fringillidae) typically provide very little; neither species’ degree of frugivory nor their phenological spread (i.e., proportion of the year spent within the community) predicts their SEF values (both *P* > 0.05). The larger species have been hunted historically and, because larger bird species typically have lower reproductive rates, will tend to be more susceptible to decline under exploitation; we therefore use −log (body mass) as a proxy for the SRF, which shows very strong phylogenetic signal (λ = 1). SEF and this SRF proxy are strongly and linearly negatively correlated (nonphylogenetic *r*^2^ = 55%; phylogenetic *r*^2^ = 41%); loss of the more susceptible 50% of the species would entail loss of all the most important species for long-distance seed dispersal (compare upper and lower histograms). This example lies in the lower right-hand corner of Figure 3, and highlights how strongly patterned and negatively correlated SEF and SRF result in loss of both function and phylogenetic diversity. Interestingly, a very similar SEF, overall seed dispersal, is much more robust to hunting: the most important species are more phylogenetically dispersed and have a wider range of SRF values (Table 1).

We applied the framework to five case studies involving plants and animals in different environmental contexts, for which species data were available on (i) either an SEF relevant to an ecosystem property or service or the species’ realized effect on it, which incorporates species abundance and which is sometimes easier to measure than SEF (see Box 4); (ii) functional traits thought likely to cause it; (iii) an SRF (or, for three of the studies, a proxy for an SRF) relevant to a well-identified environmental change; and (iv) a phylogeny for the relevant species pool. In each case study, we used a phylogenetic comparative method (phylogenetic GLS; Freckleton et al. 2002) to model how SEF or realized effect values depend on species trait values; such a method is appropriate because we wish to infer which traits underpin the SEF, so need to control for the effects of phylogenetically patterned confounding variables (Harvey and Pagel 1991). We also estimated the strength of the phylogenetic signal in the SEF or species realized effect, effect traits, and SRF, using Pagel's λ statistic (which ranges from 0 for phylogenetic randomness to 1 for strong signal; Pagel 1999; Freckleton et al. 2002) to quantify signal strength for continuous variables, and Fritz and Purvis's (2010) D (here, expressed as 1−D to put it on the same scale because D is a measure of phylogenetic dispersion rather than concentration) for binary variables. Finally, we assess the correlation across species between SEF and SRF, both phylogenetically and nonphylogenetically (as discussed above). The results from the case studies are in Table 1. Figures 4 and 5 depict two of the five examples, for which species’ SEF values were available. The left-hand side of each figure shows the phylogeny of the species in the local species pool; labeled clades are referred to in the text. Species-specific values for the effect traits and SEF are proportional to the sizes of the filled squares. Species’ SRF values are on a continuous scale, but, to aid presentation, we have split species into two equal-sized groups; the species more able to persist in the face of a particular driver are shown with large symbols, those less able to persist with small. The last column shows the SEF values of those species more able to persist. The distribution of SEF values among species is shown in the upper histogram (“SEF before filter”); the lower histogram (“SEF after filter”) shows the distribution of SEF values among the species better able to persist. Comparing these histograms shows the change in distribution of SEF values expected in response to the driver. Marked changes in the SEF distribution in the postfiltering set of species are interpreted as likely to cause changes in the ecosystem property or benefit in question. For simplicity, we did not consider in these five examples the influence that any new additions to the local species pool might have on the distribution of SEFs; we come back to this general topic in Box 4.

**Table 1:** Summary of results from five case studies

System and data references	SEF	Phylogenetic signal in SEF (λ)	SRF	Phylogenetic signal in SRF	Phylogenetic correlation of SEF and SRF, r^2^	Nonphylogenetic correlation of SEF and SRF, r^2^	Risk of loss of specified function under this driver
45 species in the Sheffield flora, Northern England (Figure 4) (Thompson et al. 1993; Cornelissen 1996)	Decomposability; high values needed for rapid nutrient cycling and soil fertility	Moderate (0.32)	Persistence in the seed bank; high values needed to survive under frequent aboveground disturbance associated with agricultural intensification	None (1−D = 0.13)	<0.001	<0.001	Very low
24 species in the Central Argentina flora (Funes et al. 1999; Pérez-Harguindeguy et al. 2000)	Decomposability; high values needed for rapid nutrient cycling and soil fertility	Strong (0.70)	Persistence in the seed bank; as above	Moderate (1−D = 0.25)	<0.001	0.027	Low
345 North-Central and West African mammal species (Fa et al. 2005; Schipper et al. 2008; Jones et al. 2009)	Bushmeat harvest rate; high values support provision of key ecosystem benefit (high-protein food) to local people	Weak (0.22)	Harvesting tolerance (indicated by intrinsic rate of population growth, r_max_, calculated from life-history data); high growth rates associated with greater tolerance	Strong (λ = 1.00)	<0.001	0.018	Low
33 Mediterranean bird species (Figure 5) (Jordano et al. 2007 and P. Jordano and J. Bascompte's unpubl. data)	Long-distance seed dispersal; high values support natural patch recolonization by woody plants of socioeconomic importance	Strong (1.00)	Hunting tolerance (indicated by small body size); small body size associated with high tolerance	Strong (λ = 1.00)	0.42	0.57	High
33 Mediterranean bird species (Jordano et al. 2007 and P. Jordano and J. Bascompte's unpubl. data)	Overall seed dispersal; high values needed for successful natural regeneration of woody plants of socioeconomic importance	Moderate (0.39)	Hunting tolerance (indicated by small body size); as above	Strong (λ = 1.00)	0.001	0.02	Very low

SEF gives the specific effect function being studied and identifies the part of the SEF distribution that supports the relevant ecosystem property. SRF gives the specific response function being considered and, for the three examples for which direct SRF data were not available, our proxy for it. In each case, SRF and SEF values were taken from the publicly available data sets listed in the first column; brief justifications for each are given in the second and fourth columns. Phylogenetic signal strength was estimated using λ (Pagel 1999) for continuous variables and D (Fritz and Purvis 2010) for binary variables (reported as 1 – D to place it on the same scale as λ). We report both phylogenetic and nonphylogenetic correlation between SEF and SRF as explained in the text. The risk of loss of function reflects both the strength and the sign of the correlation between SEF and SRF. As described in Box 2, strong phylogenetic or nonphylogenetic correlations between an SEF and SRF suggest high risk of function loss. More details of the statistical analyses are given in the main text. Plant phylogenies were created using Phylomatic (Davies et al. 2004; Webb et al. 2008) and Stace's flora (Stace 1997); the mammal phylogeny comes from Fritz et al. (2009), and the bird phylogeny from ref. (Rezende et al. 2007) with updated information from P. Jordano and J. Bascompte (pers. comm.).

From our examples it appears that phylogenetic signal in SEFs, realized effects, and SRFs may be common, as it is in the functional traits themselves. Signal was often strong in SRFs. This could be a general tendency – the physiological tolerances that determine the limits of where species persist may be more slowly evolving traits (Wiens and Donoghue 2004; Crisp et al. 2009), as are many life-history traits that underpin populations’ ability to compensate for extra mortality by increasing recruitment (Blomberg et al. 2003) – but we caution that the SRF measures showing strong phylogenetic signal in Table 1 are only proxies: more direct measures of tolerance might show much weaker phylogenetic signal. However, some published analyses show strongly patterned responses to particular drivers (Lessard et al. 2009; Fritz and Purvis 2010; Helmus et al. 2010; Guenard et al. 2011). If strong phylogenetic patterning in SRFs does turn out to be common, it would imply that much phylogenetic diversity is potentially at risk within many systems, with drivers of change tending to remove whole clades from local species pools. Such a scenario would reduce the phylogenetic redundancy of SEF provision, even if the range of SEFs is not strongly affected; this lowering of phylogenetic redundancy could reduce a system's resilience to future drivers of change (Cavender-Bares et al. 2009; Faith et al. 2010; Matthews et al. 2011). Even if SRFs are often strongly phylogenetically patterned, it is possible that strong correlations with SEFs are the exception rather than the rule, except when the same traits underpin both response and effect traits (Suding et al. 2008): if SEFs and SRFs are generated by different suites of traits that have been evolving separately, phylogenetic correlations between them are likely to be weak, although nonphylogenetic correlations could still often be strong because of the similarity of close relatives (Felsenstein 1985). If this speculative hypothesis receives support from further studies, it would suggest that ecosystem functionality may prove to be more resistant to some drivers – those unlinked to effect traits – than had been expected.

More empirical studies – especially using direct information on SRFs – are needed to see where most systems fall in the scenario matrix of Figure 3. The considerations above prompt a series of tractable research questions, listed in Box 3.

**Box 3. Combining Functional Ecology and Evolution to Assess Ecosystem Function Vulnerability: Six New Research Questions**

1. What phylogenetic patterns are most common in SEFs and SRFs? How often do they tend to show strong phylogenetic signal? What are their short-term rates of evolution? Are SRFs more phylogenetically conserved than SEFs? Do traits and effect functions with clear adaptive value (such as relative growth rate) show different patterns from those without or with less clear adaptive value (such as decomposability)?
2. How do SEFs and SRFs relate to underlying species effect and response traits? Can any generalities be drawn about groups of traits that tend to be particularly conserved or labile?
3. How commonly do SEFs correlate strongly and negatively with SRFs? Such correlations automatically ring alarm bells that the range of SEFs present in the species pool could be reduced by the corresponding driver.
4. Do different drivers of change systematically differ in the degree of phylogenetic signal in their SRFs? Drivers associated with strongly patterned SRFs are most worrying, from the perspective of ecosystem function and evolutionary capital, because of their potential to remove entire clades from the system.
5. Are particular combinations of SEF and SRF values repeatedly associated with subclades having unusual variability or diversification history? Do small-range endemics, taxa at risk of extinction, or other critical elements of the biota consistently occupy particular places along SEF or SRF distributions?
6. How do particular combinations of SEF and SRF map onto complex networks of ecological interactions? Are high SEF values consistently located at the core of these networks? Do they consistently correlate negatively with SRFs, such that their loss may entail the collapse of the whole network? Or are core species characterized by broad tolerances (high SRFs)? How are SEF and SRF distributions divided up among specific modules (sets of more strongly interacting species) within the networks?
7. Can we apply the phylogeny of SRFs and SEFs to better predict which species from the local or regional species pool are likely candidates to become invasive newcomers detrimental or beneficial to the properties of a given ecosystem subject to environmental change (see Box 4)?

## Discussion

By merging functional trait ecology and phylogeny, our approach contributes to the prediction of environmental change impact on ecosystem properties and their benefits or detriments to people. It combines a phylogenetic perspective on both species’ ecosystem-level effects (SEFs) and their responsiveness to changing environmental drivers (SRFs); vulnerabilities of ecosystem properties and benefits then depend on how SRFs and SEFs align across species and through phylogeny. The persistence of populations depends on SRFs: the likelihood that species will be lost from the local species pool depends not only on the nature and severity of environmental change but also on the inherent tolerances of individual species to those changes. However, the future of ecosystem properties and their positive or negative societal consequences depends on the SEFs of the tolerant species. It is, therefore, the degree to which SRFs and SEFs covary among the species forming the local species pools that forms the important link for predicting the impact of environmental change on ecosystems’ potential to provide functionality and benefits.

Combining comparative approaches from evolutionary ecology with ecosystem ecology is not a panacea for predicting all the myriad ways in which ecosystems will respond to environmental change. For example, we have not considered the potential role of phenotypic plasticity (Marchin et al. 2009; Chevin et al. 2010), intraspecific genotype variability (Whitham et al. 2006), or rapid in situ evolution (Hendry et al. 2008, 2010; Bassar et al. 2010) in altering the correlation between SEFs and SRFs in the face of a changing environment. Similarly, because our framework focuses on the *potential of a source pool of species* to provide ecosystem properties and benefits, we have not considered the effects of local community-level dynamics (Suding et al. 2008; Webb et al. 2010; Srivastava et al. 2012) or other processes operating at different spatial scales. These and other processes that our framework acknowledges but does not yet incorporate are summarized in Box 4.

**Box 4. What our framework does not yet consider**

**Local abundance**

We have developed our framework focusing on the potential of a source pool of species to provide ecosystem properties and benefits to people. However, species’ relative abundances, distributions, and interactions will usually modulate the realization of such potential strongly. The mass ratio hypothesis (Grime 1998; Garnier et al. 2004) suggests that species affect ecosystem properties in proportion to their local biomass. There is growing evidence that the same is true for some major ecosystem benefits, such as those relating to the regulation of the cycles of carbon, water, and major mineral nutrients, as well as trophic transfer and climate regulation (reviewed in, e.g., Chapin et al. 2000; Díaz et al. 2007). In the context of our framework, this implies that the community-level contribution to ecosystem properties and their benefits to people – that is, the realized effect – should be the product of the distribution of the SEFs among species in the species pool and each species’ abundance in the established community (Suding et al. 2008). Therefore, the loss of an abundant species that plays a dominant role in a given ecosystem property or benefit should have a larger, more immediate effect than removal of a rare species, even if the two species have the same SEF, simply because the former has a larger realized effect than the latter. However, some ecosystem effects may scale exponentially or asymptotically rather than linearly (Díaz et al. 2007), and others will show critical thresholds. For example, in some Pacific islands, populations of flying foxes are periodically reduced by hurricanes to a point beyond which, although the future of the population is not in danger, their capacity to disperse the seeds of certain big-seeded trees decreases dramatically (McConkey and Drake 2006). Other ecosystem properties or societal benefits depend more on there being a large *range* of SEF values among the species; for example, the provision of habitat for nesting, roosting, and feeding for different animals is likely to increase with a combination of different tree canopy architectures and bark textures (Nicolai 1986; Pasinelli and Hegelbch 1997). A further set of ecosystem societal benefits depend on the presence – but not necessarily the abundance – of particular species or trait values; examples include symbolic value or the presence of a chemical that may support drug development (Faith et al. 2010; Díaz et al. 2011).

Realized effects of species may be easier to measure than SEFs in some systems. Two of our examples in Table 1 – African bushmeat and overall seed dispersal by Spanish birds – are of this type. Although both these realized effects are strongly shaped by species abundance, they show some phylogenetic signal, perhaps in part because abundance itself is commonly similar among closely related species (Freckleton et al. 2002).

**Compensatory increase, colonization, and invasion**

A further complication in trying to predict the loss of potential to deliver ecosystem-level function is the compensatory increase in the abundance of rare species from the same species pool, or colonization by native species from the surrounding region or by exotic species. As SRFs and SEFs need not be tightly coupled, colonizing species may have SEF values very different from those of extirpated species. The new SEF profile in the local species pool, and therefore the potential to deliver an ecosystem property or benefit, would ultimately depend not only on the lost SEFs but also on the SEFs of the new colonists. Biological invasions provide dramatic examples of systems where large changes in ecosystem properties are brought about not necessarily by the loss of species, but rather by the spread of species carrying “new” SEF values (i.e., not present in the resident species pool). For example, the replacement of native vegetation with nitrogen-fixing invasive trees in Hawaii increases decomposition rates and the nutrient and water content of the canopy (Asner and Vitousek 2005; Hughes and Uowolo 2006; Asner et al. 2008), the spread of tussock grasses in Mediterranean Spain increases flammability (Grigulis et al. 2005), the invasion of burrowing earthworms reduces the thickness of the litter layer in North American forests and thereby select against late-successional plant species (Peltzer et al. 2010). In New Zealand, the ship rat could hardly differ more strongly from some nearly extinct endemic birds in tolerance to the disturbance regime brought about by European colonization and globalized trade (SRF); however, this invasive species appears to partially compensate for their role in pollinating native brush-inflorescence species (SEF) (Pattemore and Wilcove 2012). We have focused here mostly on losses of function through losses of species already present in an ecosystem, but our framework should also facilitate analysis of how different potential invaders in a regional species pool may alter the provision of ecosystem benefits to people.

**Intraspecific variability in functional effect and response traits**

As presented here, our framework does not explicitly incorporate within-species variability into SEFs and SRFs. In practice, intraspecific variation in the underlying functional effect and response traits will mean that conspecific individuals may differ in their SEF and SRF values. For example, intraspecific variation in fish feeding morphology causes changes in stream invertebrate and algal biomass (Palkovacs et al. 2009), whereas intraspecific variation in body mass, litter size, and sexual maturity age of mammals affects their vulnerability to extinction (González-Suárez and Revilla 2013). In plants, although trait variation among species explains the largest fraction of overall trait variation in the most comprehensive plant trait data set in existence, intraspecific variation is also substantial (Kattge et al. 2011) and in some cases is comparable with (Albert et al. 2010; Messier et al. 2010) or larger than (Grimshaw and Allen 1987; Clark et al. 2004) interspecific variation. The magnitude of within-species variation relative to among-species variation is likely to be higher when a more tightly defined clade is considered (e.g., sedges vs. all angiosperms) and will vary among functional traits (e.g., leaf dry matter content is less variable than leaf nitrogen content; Garnier et al. 2001). A measured level of variability could be accommodated by replacing each species’ SEF or SRF value with a distribution centered on that value (e.g., Felsenstein 2008). However, it would be harder to accommodate contingent plasticity (e.g., a directional response to the loss of another species) within standard comparative frameworks because trait changes are assumed not to depend on the trait distribution within an assemblage or clade. This limitation makes little practical difference at present, given the paucity of data on functional trait variation within species.

**Contemporary evolution**

In its present form, our framework considers the SRFs and SEFs of any given species as homogeneous and constant. However, species are continuously – and sometimes rapidly – evolving in response to environmental change (Hendry et al. 2008, 2010). Therefore, the mean value and the distribution of their SRFs and SEFs should also be expected to be under continuous change. For example, in response to the invasion of Australian forests by the balloon vine (*Cardiospermum grandiflorum*) from tropical America, the seed-feeding soapberry bug (*Leptocoris tagalicus*) seems to have swiftly evolved longer mouthparts that allow it to feed on – and therefore facilitate control of – this serious environmental weed (Carroll et al. 2005). Trinidadian guppies (*Poecilia reticulata*) show evolutionary changes in morphology, life history, and diet after approximately a decade of exposure to low or high predation rates. These adaptive changes in turn affect ecosystem-level properties, such as algal and invertebrate biomass and leaf decomposition, in a matter of weeks (Bassar et al. 2010). Such changes in SRFs or SEFs could be incorporated relatively easily within our framework by adding terms for genetic variation in and selection on the underlying functional traits. A number of different existing approaches could be adapted to this end, such as those of Collins and Gardner (2009) and Johnson et al. (2009). However, loss of species that fail to adapt from a system will change the selective landscape for those that remain in ways that will be hard to predict or parameterize.

**Trait-mediated biotic interactions**

In diverse assemblages of interacting species, such as plant–pollinator systems, the overall interaction pattern might be explained by the influences of the phylogenetic history of either group (Ives and Godfray 2006; Jordano 2010). Two main forces driving coevolution in such assemblages – complementarity of functional traits among interacting species and convergence of traits among species in each trophic level – pivot on species-specific traits that mediate interactions (Thompson 2005). These functional traits therefore define SEFs, and can be linked in a variety of ways to SRFs, such that removal of one species from a system may have far-reaching consequences. For example, only large frugivores are able to use the fruits of large-seeded tropical trees and effectively assist in their long-distance dispersal (da Silva and Tabarelli 2000; Forget et al. 2007). Therefore, the interactions of species and the biotic environment are mediated by specific functional traits (i.e., body mass, seed size) that are often phylogenetically patterned: closely related species tend to interact with mutualistic assemblages of similar composition (Rezende et al. 2007; Gómez et al. 2010), and functional traits can restrict the range of species with which a potential mutualist can interact. Because the loss of species from mutualistic networks can lead to cascades of nonrandom species loss (Rezende et al. 2007), the vulnerability of mutualisms to environmental drivers should be at least partially a function of the codependence of the phylogenetic structuring of SEFs and SRFs in the different trophic levels. Large-scale mutualism will therefore make the job of predicting ecosystem vulnerability more complex than for systems in which SEFs are delivered by species acting alone.

**The homology and liability of function**

Although it is the correlation between SEF and SRF values across the species in a system that is the primary concern, we also advocate mapping SEFs and SRFs onto the phylogeny of the local complement of species as part of a risk assessment. Integrating functional traits into SEFs and SRFs is valuable because analogous – as opposed to truly homologous – traits may function in subtly different ways (Pausas et al. 2004), producing different SEF or SRF values. For example, although C4 grasses are commonly treated as a single functional type, members of separately evolved lineages of C4 grasses have different SRFs in the face of elevated CO_2_ (Kellogg et al. 1999). Similarly, levels of carbonic anhydrase vary considerably among C4 plants, which therefore have different potential to contribute to global carbon flux, an SEF (Gillon and Yakir 2001; Edwards et al. 2007). Such examples suggest that, in the absence of deeper functional knowledge, there may be value in preserving as many separately evolved instances of a functional trait as possible. Not only will this help to ensure the maintenance of hidden, potentially significant functionality, but it will also buffer against further loss if separate clades are more likely to have different SRFs.

## Concluding Remarks

Our conceptual framework provides a new way to examine ecosystem service security under environmental change, by integrating species traits related to the loss of ecosystem functions with those related to persistence. Improved predictions result from considering how SEFs and SRFs relate to each other and phylogeny. Our empirical examples illustrate the utility of the approach, which will need to be more widely applied in order to make meaningful generalizations (Box 3). Will SEFs and SRFs tend to be correlated only weakly, or in potentially dangerous ways? What are the general conditions and scales at which ecosystems will tend to be especially safe or especially vulnerable?

The particular ways in which SEF and SRF are evolutionarily “achieved” in different lineages may not matter when the sole focus of interest is on how different organisms influence present-day ecosystem properties and their benefits to people. However, they matter both theoretically and practically when the aim is to analyze the origin and fate of ecosystem functionality. By analyzing comparative data on *functions,* as well as their underlying traits, evolutionary biology can join the quest for ways to manage ecosystems, and the benefits that they provide, in the face of changing environmental drivers. Similarly, for functional ecology, the crucial issue for the study of present function will often be whether response and effect traits are coupled or not (Suding et al. 2008; Lavorel and Grigulis 2012; Luck et al. 2012; see also Boxes 1 and 2), rather than phylogenetic patterns.

The ways in which people directly or indirectly, deliberately or unintendedly select for organismal traits in trying to obtain services from ecosystems are now an active focus of interdisciplinary science (Díaz et al. 2011). Evolutionary considerations, however, have been mostly ignored (Faith et al. 2010; Norberg et al. 2012). Our framework contributes to filling such gap, by offering a new synthesis integrating in a single, coherent framework the evolution of organismal traits, ecosystem process and services, and their vulnerability in the face of specific factors of environmental change. Much work remains to be done to refine, expand, and apply this conceptual framework to the assessment of ecosystem benefits to societies, conservation biology, and invasion ecology; but we see it as a step in forging fruitful linkages between ecosystem science and evolutionary biology to assess the risk of losing ecosystem properties and benefits as environments change.

## Conflcit of Interest

None delcared.

## Supporting Information

**Table S1.** Links between functional traits, species-specific functions, ecosystem properties and societal benefits, and environmental change drivers. Examples of ecosystem effect traits underpinning different Specific Effect Functions (SEFs) and response traits underpinning different Specific Response Functions (SRFs).
