# Supplementary material for: Functional traits, the phylogeny of function, and ecosystem service vulnerability
Source: Ecol Evol. 2013 Jul 30;3(9):2958–75. doi: 10.1002/ece3.601 (PMC3790543; doi:10.1002/ece3.601)
Supplement: Supplementary file 1 [file ece30003-2958-SD1.docx]

**SUPPLEMENTARY INFORMATION**

**Table S1.** **Links between functional traits, species-specific functions, ecosystem properties and societal benefits, and environmental change drivers.** (a) Examples of ecosystem effect traits (left) underpinning different Specific Effect Functions (SEFs; center), which in term influence ecosystem properties and their benefits to people (right). (b) Examples of response traits (right) underpinning different Specific Response Functions (SRFs; center), which in turn determine tolerance or susceptibility to potential environmental change drivers (right). References in (B) refer to links between response traits and environmental drivers, not necessarily to global change. See text for overlaps between effect and response traits and links between SRFs and SEFs. For SEFs, further examples are in De Bello et al. 2010. Bello et al. 2010.

**(a)**

| **Underlying functional effect traits** | **SEF** | **Some ecosystem properties and their benefits to people** | **References** |
| --- | --- | --- | --- |
| Leaf, root and stem toughness, dry matter, N, P and lignin content, specific leaf area (leaf surface displayed per unit mass) | Litter decomposability | Nutrient availability, soil fertility for the production of useful plants (e.g., fodder, wild and cultivated food and fiber plants) | ([Melillo et al. 1982](#_ENREF_77); [Hobbie 1992](#_ENREF_56); [Cornelissen 1996](#_ENREF_22); [Cornwell et al. 2008](#_ENREF_23); [Fortunel et al. 2009](#_ENREF_37)) |
| Specific leaf area, leaf N and P content, leaf mass fraction (allocation of biomass to leaves vs. to other organs | Plant potential relative growth rate | Primary productivity, fodder and fuel provision | ([Grime & Hunt 1975](#_ENREF_50); [Lambers & Poorter 1992](#_ENREF_66); [Garnier et al. 2004](#_ENREF_42)) |
| Plant height, root depth, leaf area, leaf phenology, leaf mass fraction, structure of vascular system | Plant transpiration | Ecosystem hydrological regulation including runoff, climate buffering | ([Reich 1995](#_ENREF_94); [Brauman et al. 2007](#_ENREF_13); [Chapin et al. 2008](#_ENREF_18); [Chave et al. 2009](#_ENREF_19); [Kagawa et al. 2009](#_ENREF_63)) |
| Shoot aggregation structure, cell wall to protoplast volume ratio, relative volume of hyaline cells | Bryophyte water retention capacity | Ecosystem hydrological regulation including runoff, water runoff regulation, thermal insulation (permafrost maintenance) | ([Hayward & Clymo 1982](#_ENREF_53); [Proctor 1982](#_ENREF_93); [Beringer et al. 2001](#_ENREF_10)) |
| Leaf, root, shoot and bark toughness, nutrient, lignin and polyphenol content, leaf accessibility (e.g. size and density of thorns) | Plant palatability to herbivores | Trophic transfer, food for important animals | ([Belovsky et al. 1991](#_ENREF_9); [Grubb 1992](#_ENREF_51); [Owen-Smith et al. 1993](#_ENREF_83); [Vourc'h et al. 2001](#_ENREF_107); [Bardgett & Wardle 2003](#_ENREF_8)) |
| Twig diameter and architecture, leaf dry matter content, leaf size, retention of standing dead tissue, oil and resin content | Plant flammability | Capacity of vegetation to ignite and propagate fire, soil fertility, land suitability for human settlement | ([Lavorel & Garnier 2002](#_ENREF_68); [Bond & Keeley 2005](#_ENREF_12); [Grigulis et al. 2005](#_ENREF_47); [Scarff & Westoby 2006](#_ENREF_96)) |
| Body size, mobility-related traits, defecation pattern, burrowing behaviour, solitary versus colonial habit | Animal nutrient transport capacity | Spatial redistribution of nutrients, with consequences for carrying capacity for important animals and for landscape heterogeneity | ([Owen-Smith 1988](#_ENREF_82); [Bakker et al. 2006](#_ENREF_7); [Aptroot et al. 2007](#_ENREF_4)) |
| Body size, traits of mouth parts, legs, fins or wings, pack or solitary hunting, use of hunting tools (e.g. spider webs) | Animal capacity to catch prey | Protection of primary productivity by controlling herbivores | ([Macdonald 1983](#_ENREF_71); [Post et al. 1999](#_ENREF_92); [Christiansen & Wroe 2007](#_ENREF_21)) |
| Body size, tongue and wing length and shape, body hair location and density, ability to detect different shapes, colours or scents, diurnal vs. nocturnal lifestyle | Animal pollination capacity | Enhancement of seed and fruit production for animals and people; maintenance of genetic stock diversity of plant populations. | ([Waser & Ollerton 2006](#_ENREF_108); [Kremen et al. 2007](#_ENREF_65); [Anderson et al. 2011](#_ENREF_3)) |
| Body size, mobility-related traits, gut passage time, defecation pattern, skin surface traits (e.g. hair type and density) | Vertebrate seed dispersal capacity | Enhancement of connectivity among fragmented patches; colonization of new habitats by useful plants, vegetation restoration after disturbances (e.g. fires, landslides, hurricanes) | ([Couvreur et al. 2004](#_ENREF_25); [McConkey & Drake 2006](#_ENREF_76); [Correa et al. 2007](#_ENREF_24); [Jordano et al. 2007](#_ENREF_62)) |

**(B)**

| **Underlying functional response traits** | **SRF** | **Some environmental change drivers** | **References** |
| --- | --- | --- | --- |
| Leaf area, specific leaf area, plant height, xylem vessel size and structure, leaf mass fraction, leaf phenology, root depth, epidermal thickness, stomatal traits | Plant resistance to drought | Decreased annual precipitation, increased variance in precipitation events, increased frequency of heat waves | ([Givnish 1988](#_ENREF_44); [Reich 1995](#_ENREF_94); [Maherali et al. 2004](#_ENREF_72); [Chave et al. 2009](#_ENREF_19); Zanne et al. 2010; Choat et al. 2012) |
| Bark thickness, position of dormant buds, seed coats, serotiny, germination stimulation by smoke, belowground carbohydrate storage | Plant resistance to fire | Increased frequency of fires | ([Lamont & Runciman 1993](#_ENREF_67); [Pausas et al. 2004](#_ENREF_87); [Bond & Keeley 2005](#_ENREF_12); [Paula & Pausas 2006](#_ENREF_86)) |
| Seed size and shape | Plant tolerance to high frequency and intensity of aboveground disturbance | Deforestation, agricultural intensification | ([Thompson et al. 1993](#_ENREF_103); [Moles & Westoby 2004](#_ENREF_78)) |
| Specialized stem and leaf structures for floating (e.g. *Victoria* leaves, *Salicornia* and *Typha* stems) and coping with anoxia (*Taxodium* pneumatophors), seed and fruit impermeable, floating or anchoring (e.g. *Avicennia*), root architecture (for anchoring), capacity for resprouting from fragments (e.g. *Salix*) | Plant flood tolerance | Increased variance in precipitation events, sea level rise, increased runoff in watersheds as a result of deforestation | ([Lopez 2001](#_ENREF_70); [Finlayson 2005](#_ENREF_36); [Glenz et al. 2006](#_ENREF_45); [Mommer et al. 2006](#_ENREF_79)) |
| Body size, reproductive rate, palatability, elusiveness (i.e. nocturnal, arboreal), intrinsic rate of population growth | Vertebrate tolerance to hunting | Higher demand for bushmeat, as a result of human encroachment into forests and or increased scarcity of other sources of protein (e.g. fisheries) | ([Johnson 2002](#_ENREF_59); [Wright 2003](#_ENREF_115); [Fa et al. 2005](#_ENREF_33); [Cowlishaw et al. 2009](#_ENREF_26)) |
| Body size, chemical and physiological traits, surface to volume ratio, seasonality | Invertebrate (e.g. woodlice, springtails) drought tolerance | Decreased annual precipitation, increased variance in precipitation events, increased frequency of heat waves, increased soil desiccation as a result of land use practices | ([Tsai et al. 1998](#_ENREF_105); [Makkonen et al. 2011](#_ENREF_73)) |
